# The first reported case of *Burkholderia contaminans* in patients with cystic fibrosis in Ireland: from the Sargasso Sea to Irish Children

**DOI:** 10.1186/s12890-016-0219-z

**Published:** 2016-04-22

**Authors:** Rachel F. Power, Barry Linnane, Ruth Martin, Noelle Power, Peig Harnett, Brian Casserly, Nuala H. O’Connell, Colum P. Dunne

**Affiliations:** Graduate Entry Medical School and Centre for Interventions in Infection, Inflammation & Immunity (4i), University of Limerick, Limerick, Ireland; University Hospital Limerick, Dooradoyle Limerick, Ireland

**Keywords:** *Burkholderia contaminans*, Cystic fibrosis, First incidence, Ireland, Case report

## Abstract

**Background:**

*Burkholderia contaminans* is an emerging pathogen in the cystic fibrosis (CF) setting. Included in the *Burkholderia cepacia* complex (Bcc), *B. contaminans* is a Gram negative, motile, obligate aerobe previously classified as a pseudomonad. Previous reports have described *B. contaminans* isolation from patients in Portugal, Switzerland, Spain, Argentina and the USA. This, however, is the first report relating to *B. contaminans* affecting Irish patients with CF, initially detected in a paediatric setting.

**Case presentation:**

*Burkholderia contaminans* was identified in the routine analysis of sputum from a fourteen year old boy, at his annual review and subsequently from the sputum from his 19 year old brother. *RecA* gene sequencing and pulsed field gel electrophoresis (PFGE) were unable to distinguish between the isolates, which demonstrated with susceptibility to ciprofloxacin, cotrimoxazole, meropenem, pipercillin/tazobactam and ceftazidime. Both isolates were resistant to aztreonam, with reduced susceptibility to tobramycin. Following treatment with intravenous meropenem and ceftazidime, oral ciprofloxacin and nebulised tobramycin for 6 weeks, sputum specimens from both patients were negative for *B. contaminans*. No other member of the local CF cohort proved positive.

**Conclusions:**

Bcc bacteria are associated with poor prognosis in CF and decreased life expectancy, specifically leading to a more rapid decline in lung function and, in some cases, to a fatal necrotizing pneumonia known as the “cepacia syndrome”. Some species exhibit innate resistance to multiple antimicrobial agents and their transmission rate can be high in susceptible patients. In that context, we describe the first incidence of CF-related *B. contaminans* in Ireland and its successful eradication from two patients, one paediatric, using an aggressive antimicrobial regimen.

## Background

*Burkholderia cepacia complex* (Bcc) is a grouping of bacteria comprising at least 17 species capable of infecting susceptible patients including the immunocompromised and those with cystic fibrosis (CF) [1, 2]. *Burkholderia contaminans* is one such species, first identified as a contaminant of a marine-derived nucleic acid sample obtained from the Sargasso Sea [3, 4]. *Burkholderia contaminans* is increasingly associated with CF [5–11] although not exclusively as other hospitalised non-CF patients have also been affected [9, 12–14].

Globally, Ireland has the highest prevalence of CF (a recessive autosomal disease associated with increased susceptibility to respiratory infection) with prevalence of 2.98 per 10,000 compared to European and USA prevalence of 0.737 and 0.797 per 10,000, respectively [15]. In that context, this first detection of *B. contaminans* in any Irish patient and, especially CF patients, is noteworthy. In this report, similar to other studies involving *B. contaminans*, we attempt to correlate colonisation with incidence of acute exacerbation of symptoms. In addition, we describe the outcomes of molecular typing of the isolates, using *recA* sequencing and pulsed field gel electrophoresis (PFGE) techniques successfully employed elsewhere [6, 16, 17]. As *B. contaminans* is emerging as an identifiable, discrete pathogen described previously as “virtually impossible to eradicate from the CF lung, posing a serious clinical threat” [8] and is currently without definitive guidelines for antimicrobial sensitivity/resistance, we detail our antimicrobial susceptibility testing, and the successful eradication of *B. contaminans* from both patients using an aggressive treatment regimen.

## Case presentation

In August 2013, two male CF patients, siblings aged 14 and 19 were identified as *Burkholderia contaminans* positive at University Hospital, Limerick, Ireland (where the first Irish CF-related multidrug-resistant *Bordetella petrii* was identified) [18]. Retrospective analyses of patient charts and stored sputum samples determined frequency of hospitalisation, episodes of infections, and antimicrobial use prior to *B. contaminans* detection and throughout treatment. Emphasis was placed on presence/absence of clinical features during treatment, in particular lung function, efficacy of antimicrobial therapy and patient outcome.

Genotypically, Patients A (aged 14) and B (19 years) are homozygous for the F 508del mutation (within the CF transmembrane conductance regulator causing loss of the amino acid phenylalanine, affecting chloride ion channels in cell membranes and leading to production of thickened mucus). Both resided in the family home in the Midwest of Ireland.

Between diagnosis as a neonate and August 2013, 14 year old Patient A had experienced four admissions for treatment of exacerbations of CF, complicated by reactive airways disease with persistent cough and poor compliance with his medications and adherence to physiotherapy. Prior to August 2013, his cough swabs and sputum cultures had yielded *Staphylococcus aureus*, *Haemophilus parainfluenzae*, *Candida* species and *Aspergillus fumigatus*. He had also acquired, and had been treated for, *Pseudomonas aeruginosa* in 2008, which recurred in 2012. Attempts to eradicate *P. aeruginosa* failed and he was deemed to be chronically infected in November 2012. As the strain was resistant to tobramycin, he was commenced on long-term anti-pseuodomonad therapy comprising alternate months of two mega-units of nebulised colistin twice daily with 75 mg of nebulised aztreonam three times daily, along with oral azithromycin 250 mg once daily taken three times per week. Struggling with adherence, his Forced Expiratory Volume in 1 s (FEV_1_) dropped from 62 % in August 2012 to 46 % in June 2013.

On 6th August 2013, Patient A presented for annual review. *Staphylococcus aureus* and *P. aeruginosa* were identified from his routine sputum sample. In addition, however, *Burkholderia contaminans* was also detected, representing the first such incidence in Ireland. Patient A’s FEV_1_ was 38 %, his lowest recorded. His full blood count at the time showed a mildly elevated white cell count of 10.45 10^9^/L, with a neutrophil count of 5.86 10^9^/L.

Between diagnosis as a neonate and August 2013, 19 year old Patient B had also demonstrated poor compliance with his treatment regimen, complicated by insulin dependent diabetes mellitus and pulmonary fibrosis. Like his younger brother, Patient B’s cough swabs and sputum cultures had consistently yielded varying and mixed species: *H. parainfluenzae*, *H. influenzae*, *S. aureus*, *Streptococcus pneumoniae,* and *A. fumigatus*. In 2007, he acquired *Pseudomonas aeruginosa* (tobramycin resistant) and, despite multiple attempts at eradication, he became chronically infected. His FEV_1_ fluctuated between 17 and 95 % throughout this time, with a baseline of 68 %. His antimicrobial medications included oral azithromycin 250 mg 3 days per week and two mega-units nebulised colistin twice daily, on alternate months.

On 2nd July 2013, Patient B was admitted with an infective exacerbation and was treated with a course of intravenous meropenem (2 g every 8 h) and tobramycin (480 mg once daily) combined with intense physiotherapy. He was also provided nutritional support for a body mass index of 17. Two days after detection of *B. contaminans* in Patient A’s sputum, his brother was confirmed as positive.

Microbiological analysis was comprehensive, utilising *recA* sequencing and pulsed field gel electrophoresis (PFGE). More specifically, following growth of bacteria on “*Burkholderia cepacia* specific agar” (LIP Diagnostics, Fannin Healthcare, Ireland), identification was confirmed by Matrix-Assisted Laser-Desorption/Ionization Time-Of-Flight Mass Spectrometry (MALDI-TOF MS) and samples forwarded to the Antimicrobial Resistance and Healthcare Associated Infections Reference Unit (AMRHAI, Public Health England) for confirmation by amplification of the *recA* gene using the BCR1 and BCR2 primers described previously [19]. Pulsed-field gel electrophoresis (PFGE) of *Xba*I-digested genomic DNA was also performed. Both methods indicated presence of a single discrete *B. contaminans* strain.

Currently, there is no definitive classification of *B. contaminans* as sensitive/resistant to specific antimicrobials. In this study, the efficacy of selected antimicrobials against the *B. contaminans* isolates was initially assessed using Etest® (bioMérieux, France) test strips to ascertain minimum inhibitory concentrations (MIC): aztreonam MIC >256 mg/L; meropenem MIC = 2.0 mg/L; colistimethate >256 mg/L; tobramycin = 64 mg/L; ciprofloxacin = 1 mg/L; pipercillin/tazobactam MIC >256 mg/L; and ceftazidime MIC = 8 mg/L. Both isolates were sent to the UK CF microbiology reference laboratory at the Freeman Hospital (Newcastle upon Tyne) for synergy testing (i.e., determining the optimal antimicrobial combinations to eradicate infection). A combination of tobramycin and meropenem achieved only inhibition of growth, while combinations of ceftazidime with meropenem, ciprofloxacin, and tobramycin proved bactericidal.

It was decided to adopt a 6 week quadruple treatment strategy comprising IV ceftazidime 50 mg per kg per dose every 8 h, IV meropenem 40 mg per kg per dose [to a maximum of 2 g per dose], oral ciprofloxacin 15 mg per kg per dose bd, and nebulised tobramycin 300 mg bd. However, Patient A discontinued meropenem at 3 weeks as a consequence of developing severe back and muscle pain, a reaction that was reported to the Regulatory Agency. He completed treatment with the remaining three antimicrobials and his FEV_1_ improved from 38 to 61 %. He was subsequently treated with nebulised aztreonam and colistimethate, alternating monthly for a year. Patient B successfully completed the initial regimen over 6 weeks, with similar improvement reflected in an cumulative increase in FEV_1_ of 26 % over the subsequent 1 year period, along with an increase in weight from 54 kg in July 2013 to 61.9 kg in September 2014. *Burkholderia contaminans* has been eradicated successfully from both patients, and they remain free of infection 30 months post treatment. Both patients now demonstrate increased adherence to treatment and physiotherapy, with sustained improved lung function.

At the time of *B. contaminans* detection, Patient A was attending the paediatric clinic while his brother was admitted to the adult CF clinic. The clinics are located in separate areas of the hospital. However, upon identification of the pathogen, the local Bcc infection control policy was implemented and the brothers were segregated from all other CF patients. Containment appears to have been effective as *B. contaminans* has not been detected in our hospital since.

## Conclusions

Species of the *Burkholderia cepacia* complex are associated with opportunistic infection in patients with CF, and are associated with a worse prognosis and decreased life expectancy [10]. One such bacterium is *B. contaminans* that, since first identification in 2009, has become recognized as ubiquitous in the environment [20, 21], including healthcare facilities where it has caused outbreaks [12, 14, 22]. However, the discrete impact of *B. contaminans* is poorly understood as, firstly, its incidence is typically reported as part of multi-bacterial infections [10] and, secondly, its presence may not have been identified at all pre-2009 or, since then, the species incorrectly identified using available molecular techniques [23]. Indeed, it is noticeable that recent studies of *B. contaminans* have, in many cases, focused on development of accurate and more sophisticated molecular characterization of the species including mechanisms mediating its pathogenicity [5, 24–26]. However, it is somewhat ironic that some of those studies are focused on developing novel antimicrobial/antifungal agents produced by *B. contaminans* [27–29].

In this report, we describe the first clinical incidence of *B. contaminans* in Ireland. As with others, our observation related to CF patients, one of whom was a child. Given the high prevalence of CF in Ireland, and the increasingly frequent isolation of *B. contaminans* from CF patients in some countries (e.g., [11], our reaction to identification of the pathogen for the first time was one of extreme prudence. Our empiric, and subsequently targeted, antimicrobial treatment regimen was deliberately aggressive, as was our Bcc infection control policy. Ultimately, both proved effective, resulting in eradication of the *Burkholderia* strain from the two patients and containment of the potential risk to other CF patients attending the hospital.

Further, given the current lack of defined guidelines regarding *B. contaminans* antimicrobial susceptibilities, we present our MIC data (detailing resistance to aztreonam and tobramycin and susceptibility to ciprofloxacin, cotrimoxazole, meropenem, pipercillin/tazobactam and ceftazidime) to supplement results published recently from Switzerland [30]. We hope that our descriptions of the cases and our successful treatment choices will prove useful to clinicians encountering *B. contaminans* and prove beneficial to them and their patients.

### Ethics approval and consent to participate

Ethical approval to report this case was not required.

### Consent for publication

Written informed consent was obtained from the patients and their parents for publication in this case report. A copy of the written consent is available for review by the Editor of this journal.

### Availability of data and materials

There are no additional data. The bacterial isolates involved in this case can be shared.

## Abbreviations

CF: cystic fibrosis
PFGE: pulsed field gel electrophoresis
FEV_1_: forced expiratory volume in one second
MIC: minimum inhibitory concentration
